# MUC5B levels in submandibular gland saliva of patients treated with radiotherapy for head-and-neck cancer: A pilot study

**DOI:** 10.1186/1748-717X-7-91

**Published:** 2012-06-15

**Authors:** Tim Dijkema, Chris H J Terhaard, Judith M Roesink, Cornelis P J Raaijmakers, Petra A M van den Keijbus, Henk S Brand, Enno C I Veerman

**Affiliations:** 1 Department of Radiotherapy, University Medical Center Utrecht, hp Q00.118, Heidelberglaan 100, 3584, CX, Utrecht, The Netherlands; 2 Section of Oral Biochemistry, Academic Centre for Dentistry Amsterdam (ACTA), Amsterdam, The Netherlands

**Keywords:** Xerostomia, Head-and-neck cancer, Radiotherapy, MUC5B, Mucin, Submandibular gland

## Abstract

**Background:**

The salivary mucin MUC5B, present in (sero)mucous secretions including submandibular gland (SMG) saliva, plays an important role in the lubrication of the oral mucosa and is thought to be related to the feeling of dry mouth. We investigated if MUC5B levels in SMG saliva could distinguish between the presence or absence of severe dry mouth complaints 12 months after radiotherapy (RT) for head-and-neck cancer (HNC).

**Findings:**

Twenty-nine HNC patients with a residual stimulated SMG secretion rate of ≥0.2 ml/10 min at 12 months after RT were analyzed. MUC5B (in U; normalized to 1) and total protein levels (mg/ml) were measured in SMG saliva at baseline and 12 months after RT using ELISA and BCA protein assay, respectively. Overall, median MUC5B levels decreased after RT from 0.12 to 0.03 U (*p* = 0.47). Patients were dichotomized into none/mild xerostomia (n = 12) and severe xerostomia (n = 17) based on a questionnaire completed at 12 months. SMG and whole saliva flow rates decreased after RT but were comparable in both groups. The median MUC5B level was higher in patients with no or mild xerostomia compared to patients with severe xerostomia (0.14 vs 0.01 U, *p* = 0.22). Half of the patients with severe xerostomia had no detectable MUC5B at 12 months after RT. No differences in total protein levels were observed.

**Conclusions:**

Qualitative saliva parameters like MUC5B need further investigation in RT-induced xerostomia. This pilot study showed a trend towards lower MUC5B levels in the SMG saliva of patients with severe xerostomia 12 months after RT for HNC.

## Findings

### Background

Xerostomia after radiotherapy (RT) for head-and-neck cancer (HNC) has a major impact on quality of life in HNC survivors [1,2]. Sparing of the parotid glands (PG) using intensity-modulated radiotherapy (IMRT) significantly improves parotid gland function in patients treated for HNC [3,4]. In some studies however, the use of parotid gland-sparing RT alone did not improve patient-reported xerostomia [4,5]. Probably the submandibular, sublingual and minor salivary glands play an important role in the subjective sense of moisture in between meals [6]. They secrete glycoproteins (mucins) that cover and protect the underlying mucosa (MUC5B and MUC7). The larger salivary mucin MUC5B, present in (sero)mucous secretions including submandibular gland (SMG) saliva, is thought to be related to the perception of dry mouth by retaining moisture in the mucosa [7,8].

Our hypothesis in this pilot study was that MUC5B levels, as a qualitative parameter in human saliva, could better explain xerostomia compared to quantitative saliva measurements in RT patients. For that purpose, we investigated if MUC5B levels in SMG saliva could distinguish between the presence or absence of severe dry mouth complaints 12 months after RT for HNC.

### Patients

Twenty-nine patients were selected from a larger population, included in prospective studies on salivary gland function after RT for HNC at our department [9,10]. The selected patients all had a residual stimulated SMG secretion rate of ≥0.2 ml/10 min at 12 months after RT. The amount of 0.2 ml is the threshold for MUC5B analysis in saliva.

### Saliva flow measurements

Techniques that were used for objective saliva measurements have been described previously [3,9]. Stimulated salivary flow rates were measured before treatment and at one year after RT. Citric acid solution (5%) was applied on the anterior part of the tongue every 60 seconds, for 10 minutes. Saliva near Wharton’s duct orifices in the floor of the mouth was collected by gentle suction with a micropipette, representing predominantly SMG saliva but also varying amounts from the sublingual glands (SLG). Stimulated PG saliva was collected separately using Lashley cups. After collection, saliva samples were stored at -20 °C until analysis.

### Saliva protein assays

MUC5B levels in the SMG saliva samples were determined by an enzyme-linked immunosorbent assay (ELISA) as described previously [11,12]. The monoclonal antibody F2 used for quantification of MUC5B specifically recognises the terminal part of the carbohydrate moiety, sulfo-Lewis^a^ SO_3_-3Gal_1-3GlcNAc. This structure is present on MUC5B secreted by the SMGs, SLGs and palatal (minor) salivary glands. MUC5B was quantified by comparison to unstimulated whole saliva from a pooled sample of 10 healthy staff members of a dental faculty with optimal oral health. Each study patient was compared to the pooled sample of these healthy volunteers. MUC5B levels were expressed in relative units, with the MUC5B concentration in the pooled saliva of healthy volunteers normalized to 1. 1 Unit is approximately 230 μg/ml [13].

The total protein content (mg/ml) was measured in SMG saliva using the BCA Protein Assay Reagent (Pierce, Rockford, IL, USA) with bovine serum albumin (BSA) as a standard.

### Assessment of patient-reported xerostomia

All patients completed a xerostomia questionnaire (XQ) before RT and 12 months after RT. The XQ contains questions related to xerostomia and is scored on a 5-point Likert scale. A score of ‘1’ means no complaints, while a score of ‘5’ implies complaints are always present. In this analysis, we utilized two questions addressing the sensation of dry mouth during daytime (‘Do you have a dry mouth during the day’) and nighttime (‘Do you have a dry mouth at night’). Xerostomia was dichotomized into ‘severe’ (grade 4-5) or ‘none-to-mild’ (grade 1-3). Patients who had grade 4-5 xerostomia during the day *and/or* the night at 12 months were grouped together. Patients with no or mild complaints (grade 1-3) during day- and nighttime were also grouped together in the analyses.

### Statistical analysis

Patient characteristics and MUC5B/protein levels were reported using descriptive statistics (median, ranges or proportions; where appropriate). Correlations were calculated using Pearson’s correlation coefficient (*r*). Paired samples obtained before and after RT were compared using Wilcoxon signed ranks test. Subgroup differences in saliva flow rate, MUC5B and protein levels were analyzed using the Mann–Whitney *U* test. Fisher’s Exact test was used to compare proportions within cross tabulations. All analyses were performed using SPSS version 16 (SPSS Inc, Chicago, IL, USA). A *p* value of <0.05 was considered statistically significant.

## Results

The 29 patients included in this pilot study had a mean age of 58 years (range 35-82 yr) and 22 (76%) were male. All patients had a squamous cell carcinoma of the head and neck, with 19 oropharyngeal tumors (66%), 6 laryngeal (21%), 3 nasopharynx (10%) and 1 oral cavity (3%). IMRT was used in 21 (72%) patients, the remainder was treated with conventional (2D) techniques [9]. The mean dose to the SMGs was 55.9 Gy (range 10.6-71.1 Gy).

Overall, the median MUC5B level decreased after RT from 0.12 U to 0.03 U (*p* = 0.47). MUC5B levels at 12 months showed a very weak correlation with the mean SMG dose in Gy (Pearson *r* = 0.18). The median total protein content of SMG saliva decreased slightly after treatment (1.00 and 0.82 mg/ml respectively, *p* = 0.62).

Twelve months after RT, 17 patients had severe complaints of dry mouth at day- and/or nighttime, 12 patients had no or mild complaints. The SMG and whole saliva (PG + SMG) flow rates decreased after RT but were comparable in both groups at 12 months (Table 1).

**Table 1:** Median saliva flow rates, MUC5B and total protein levels 12 months after RT in patients with and without severe xerostomia during daytime and/or nighttime

		**No/mild xerostomia**	**Severe xerostomia**	** *p* **
		(n = 12)	(n = 17)	
SMG flow rate	ml/10 min	0.69	0.80	0.66
WS flow rate (PG + SMG)	ml/10 min	3.35	2.80	0.82
MUC5B concentration	U	0.14	0.01	0.22
Undetectable MUC5B *	%	25	47	0.27
MUC5B x flow rate ^†^	μg/10 min	15.0	1.3	0.37
Total protein	mg/ml	0.87	0.82	0.76

Abbreviations: *SMG* = submandibular gland, *WS* = whole saliva, *PG* = parotid gland, *U* = units; normalized to 1 for unstimulated saliva in healthy controls.

* MUC5B level in SMG saliva equal to 0.00 U at 12 months after RT.

^†^ The MUC5B x flow rate product (in μg/10 min) was calculated by multiplying the MUC5B concentration (in U) and SMG flow rate (in ml/10 min) for each patient at 12 months after RT and assuming 1 Unit is 230 μg/ml of MUC5B [13].

No statistical differences were found in the baseline MUC5B levels between the groups (median 0.12 versus 0.14 U for the group with and without severe xerostomia, respectively) nor in the change from baseline in each individual patient (calculated as ΔMUC5B: median 0.04 versus 0.11 U respectively, *p* = 0.9). ΔMUC5B showed a small negative correlation with the SMG mean dose in Gy (Pearson *r* = -0.26).

**Figure 1:**
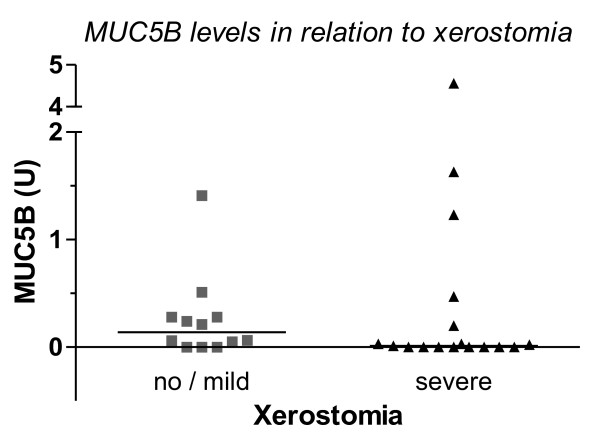
**Comparison of MUC5B levels (in U) in patients with and without severe xerostomia 12 months after RT, measured using ELISA.** The horizontal line represents the median for each group.

At 12 months, the median MUC5B was higher in patients with no or mild xerostomia compared to patients with severe complaints, although the difference was not statistically different at the 0.05 level (Table 1). The group with severe complaints was characterized mainly by undetectable MUC5B levels and a number of outliers (Figure 1). Two of the outliers represented a 14- and 123-fold increase in the MUC5B concentration from baseline. Repeating the analyses without these two extremes showed a borderline significant higher MUC5B level in the patients with no or mild xerostomia (median 0.14 vs 0.00 U, *p* = 0.055) at 12 months.

When we combined the qualitative (MUC5B in U) and quantitative (SMG flow rate in ml/10 min) measurements for each individual patient by multiplying both parameters (MUC5B x flow rate; Table 1) and assumed 1 Unit is 230 μg/ml of MUC5B [13], we found an approximate 10-fold lower value (in μg/10 min) in the group of patients with severe complaints of dry mouth, although statistical difference was not reached in this small study.

The median total protein content in SMG saliva after 12 months was similar in both groups.

### Discussion

This pilot study did not show a statistically significant difference in MUC5B levels in SMG saliva of patients with and without severe xerostomia 12 months after RT, although a trend was observed towards higher MUC5B levels in patients with fewer complaints of dry mouth. Almost half of the patients with severe xerostomia had no detectable MUC5B at 12 months after RT. The results are therefore of interest and do need investigation within a larger cohort of patients. An ongoing prospective study at our department, investigating the effect of sparing the contralateral SMG on xerostomia after RT, is expected to yield more SMG saliva samples for future qualitative analyses [10].

As both subgroups in this study had comparable amounts of saliva but differed in the severity of their complaints, a case is made for qualitative saliva parameters rather than quantitative measurements in xerostomia research. The high-molecular weight salivary mucin MUC5B contains large carbohydrate groups that are heterogeneous and include sulfated and sialylated oligosaccharides, retaining large amounts of water. The unique rheological properties of MUC5B contribute to the formation of a thin salivary film and the resulting demulcent coat is thought to hydrate and lubricate the soft tissues of the mouth [14]. Serous acinar cells found in the parotid glands do not produce mucins. The latter may explain why, in RT for head-and-neck cancer, sparing of the parotid glands alone does not seem to improve patient-reported xerostomia [4].

Apart from the free MUC5B fraction measured in SMG saliva in this study, there may be other mucin-related factors that can explain xerostomia. First, in stead of the free fraction, the amount of mucosa-bound MUC5B may better explain which patients will complain of a dry mouth. Pramanik et al. showed, that in (non-RT) dry mouth patients unable to provide a measurable unstimulated saliva sample (zero flow), MUC5B was often still present on all mucosal surfaces [15]. Therefore, mucins retained on the mucosa of dry mouth patients are presumably less hydrated than in normal subjects. In this regard, post-translational modifications of MUC5B synthesis and in particular sulfation levels (rather than mucin levels per se) could result in a reduced water content of mucins and explain the dry mouth sensation. Loss of MUC5B sulfation was observed in the mucous acini from labial salivary glands of patients with Sjögren syndrome and was unrelated to alterations in saliva quantity [7]. To what extent these findings can be extrapolated to patients with radiotherapy-induced xerostomia needs to be investigated. Third, differently glycosylated MUC5B species are present in saliva. In single glandular secretions and even in one secretory acinus different glycoforms are expressed, pointing to a large heterogeneity in mucin molecules [16]. Moreover, MUC5B from different glandular sources have different rheological properties that may influence fluid retention on mucosal surfaces [17]. In this study, a specific (sulfo)glycolysation motif (sulfo-Lewis^a^) was detected, present on MUC5B secreted by the SMGs, SLGs and palatal minor glands. Possibly, (absence of) other MUC5B glycoforms can better explain why some patients with recovering SMG secretion after RT complain of a dry mouth and others do not.

## Competing interests

The authors declare that they have no competing interests.

## Authors’ contributions

TD participated in the design of the study, performed data collection, statistical analysis and drafted the manuscript. CT, JR and NR participated in the design and coordination of the study and data collection. PvdK carried out the immunoassays and protein analyses. HB and EV participated in the design of the study and interpretation of the data, HB also assisted with the statistical analysis. All authors read and approved the final manuscript.
